# Broad funding for oxaliplatin in Ontario (finally!)

**DOI:** 10.3747/co.2007.155

**Published:** 2007-12

**Authors:** D. Jonker, J. Maroun, K. Spithoff

**Affiliations:** * The Ottawa Hospital Regional Cancer Centre, Ottawa, Ontario; † Cancer Care Ontario, Program in Evidence-Based Care, McMaster University, Hamilton, Ontario

Since the presentation of trials such as the Intergroup N9741 study (National Cancer Institute of Canada co.15) and the mosaic (Multicenter International Study of Oxaliplatin/5-fu–lv in the Adjuvant Treatment of Colon Cancer) trial at the 2003 American Society of Clinical Oncology meeting, oncologists have broadly accepted that oxaliplatin is part of the standard management of colorectal cancer, both in the metastatic and adjuvant settings 1–4.

The Gastrointestinal Cancer Disease Site Group within Cancer Care Ontario’s Program in Evidence-Based Care (pebc) immediately began the thorough process of systematic review, development of evidence-based recommendations, review by the Report Approval Panel (a methodologic oversight group), practitioner feedback, and eventual finalization and publication of guidelines. For oxaliplatin in colorectal cancer, that process was required for three settings:

- Metastatic colorectal cancer
- Resected colon cancer
- Resected rectal cancer

The guideline for the metastatic colorectal cancer setting was published in *Current Oncology* in 2006 5. The guideline for stages ii and iii colon cancer was updated to incorporate the most recent data for oxaliplatin (Cancer Care Ontario, Program in Evidence-Based Care. Evidence-based series 2–29: Adjuvant systemic chemotherapy for stage ii and iii colon cancer following complete resection. In development). Final approval and publication of the stages ii and iii colon and rectal cancer guidelines are pending (Cancer Care Ontario, Program in Evidence-Based Care. Evidence-based series 2–3, Ver. 2.2007. Postoperative adjuvant radiotherapy and/or chemotherapy for resected stage ii or iii rectal cancer. In development).

Once a guideline is made available by the pebc, it undergoes a joint review process in Ontario that evaluates all cancer-related drugs for consideration under the New Drug Funding Program or the Ontario Drug Benefit program, or both. The Committee to Evaluate Drugs (ced), an expert advisory committee to the Ministry of Health and Long-Term Care, conducts the joint review and makes a recommendation regarding the funding of the drug in the province of Ontario 6.

Given the intrinsic delays in the rigorous pebc guideline development cycle, the ced has always accepted late drafts of the recommendations to assist in making timely decisions for emerging therapies. The guidelines on colorectal cancer were therefore made available to the ced in 2006.

Complicating the story for oxaliplatin was the absence of a Health Canada Notice of Compliance (noc). In the context of the incomplete intellectual property protection pertaining in Canada before October 2006, Sanofi-Aventis could not submit its confidential data 7 from an extensive research and development program without running the risk of seeing its oxaliplatin product Eloxatin immediately become genericized. When that situation changed, and data protection provisions were read into law, Sanofi-Aventis could then apply for 8 years of patent protection under the new legislation.

And so, while the rest of the world’s regulators approved oxaliplatin (the U.S. Food and Drug Administration’s approval for metastatic disease came in colon cancer in 2004 2002, and for stage iii^8^), Health Canada could make oxaliplatin available only through a special access program. Canadians were left with a patchwork of provincial approaches to the coverage of oxaliplatin. British Columbia provided the drug; Quebec gave approval only on a hospital-by-hospital basis. Ontario took the rigid position that, as long as the drug lacked a noc, it could not be funded. Ontario patients were at the mercy of the self-pay, albeit generously Sanofi–Aventis-sponsored, Special Access Program—a means-tested co-pay model.

On June 15, 2007, oxaliplatin received a noc for metastatic colorectal cancer, and the ced quickly reviewed the drug. The funding conditions announced (Table I) in Ontario represent the most widespread inclusive coverage for oxaliplatin of any province. Although approval was long in coming, Ontario patients and their oncologists now have one less stressful issue to discuss during that first consultation: the financial issues surrounding oxaliplatin as standard care.

## Figures and Tables

**TABLE I:** Oxaliplatin approval in colorectal cancer

Clinical setting	U.S. Food and Drug Administration	Health Canada Notice of Compliance	cco pebc recommendations	Ontario CED decision
Colorectal cancer				
1st-Line metastatic	“Use in combination with infusional 5-fluorouracil (5-fu) and leucovorin (lv) for the initial treatment of advanced colorectal cancer.” January 2004	“Use in combination with 5-fluorouracil (5-fu) and leucovorin (lv) as treatment of patients with metastatic colorectal cancer.” June 15, 2007	folfox4, mfolfox6, or xelox June 2005	Funded (folfox regimens at 85 mg/m^2^)
2nd-Line metastatic (for example, post-folfiri)	Accelerated approval: “For use in combination with infusional 5-fu/lv for the treatment of patients with metastatic carcinoma of the colon or rectum whose disease has recurred or progressed during or within six months of completion of first-line therapy with the combination of bolus 5-fu/lv and irinotecan” August 9, 2002		folfox4, mfolfox6, or xelox June 2005	Funded (folfox regimens at 85 mg/m^2^)
Colon cancer				
Stage iii	“Use in combination with infusional 5-fluorouracil/ leucovorin (5-fu/lv) for adjuvant treatment of stage iii colon cancer patients who have undergone complete resection of the primary tumor” November 2004	No indication at present	Recommendation pending	Funded (folfox or flox regimens at 85 mg/m^2^)
Stage ii	No indication at present	No indication at present	Recommendation pending	Funded (folfox or flox regimens at 85 mg/m^2^)
Rectal cancer				
Stage ii/iii	No indication at present	No indication at present	Recommendation pending	Funded (folfox or flox regimens at 85 mg/m^2^)

cco pebc = Cancer Care Ontario, Program in Evidence-based Care; ced = Committee to Evaluate Drugs; folfox = 5-fluorouracil/leucovorin/oxaliplatin; xelox = capecitabine/oxaliplatin; folfiri = 5-fluorouracil/leucovorin/irinotecan; flox = bolus 5-fluorouracil/leucovorin/oxaliplatin.
